# Characterization of the putative yeast mitochondrial triacylglycerol lipase Tgl2

**DOI:** 10.1016/j.jbc.2025.108217

**Published:** 2025-01-23

**Authors:** Vitasta Tiku, Zacharias Fakih, Takashi Tatsuta, Martin Jung, Doron Rapaport, Kai Stefan Dimmer

**Affiliations:** 1 Interfaculty Institute of Biochemistry, University of Tübingen, Tübingen, Germany; 2 Max Planck Institute for Biology of Ageing, Köln, Germany; 3 Medical Biochemistry and Molecular Biology, Saarland University, Homburg, Germany

**Keywords:** mitochondria, intermembrane space, lipids, lipases

## Abstract

Mitochondria derive the majority of their lipids from other organelles through contact sites. These lipids, primarily phosphoglycerolipids, are the main components of mitochondrial membranes. In the cell, neutral lipids like triacylglycerides (TAGs) are stored in lipid droplets, playing an important role in maintaining cellular health. Enzymes like lipases mobilize these TAGs according to cellular needs. Neutral lipids have not yet been reported to play an important role in mitochondria so the presence of a putative TAG lipase–Tgl2, in yeast mitochondria is surprising. Moreover, *TGL2* and *MCP2*, a high-copy suppressor for ER mitochondria encounter structure deficient cells, display genetic interactions suggesting a potential link of both proteins to lipid metabolism. In this study, we characterize in detail Tgl2. We show that Tgl2 forms dimers through intermolecular disulfide bridges and a cysteine-dependent high molecular weight complex. Furthermore, we could identify the lipase motif and catalytic triad of Tgl2 through *in silico* comparison with other lipases. Mutating each of the three catalytically active residues resulted in variants that failed to rescue the growth phenotype of *mcp2*Δ *tgl2*Δ double deletion strain supporting the assumption that these residues are indeed essential for the protein’s function. Additionally, we discovered that the catalytically active aspartate residue (D259) is important for protein stability. Steady state level analyses with unstable variants of Tgl2 led to the identification of Yme1 as the protease responsible for its quality control. Finally, we provide evidence that the overall increase in TAGs in cells lacking Mcp2 and Tgl2 originates from the mitochondria. Collectively, our study provides new insights into a key player in mitochondrial lipid homeostasis.

Mitochondria are complex organelles with a wide array of functions. Their endosymbiotic bacterial ancestry has enabled them to evolve into semiautonomous, bilayered entities consisting of an outer (mitochondrial outer membrane, MOM) and an inner mitochondrial membrane (mitochondrial inner membrane, MIM) (1, 2). These membranes are distinct in their protein content and lipid composition, separating the organelle into two aqueous subcompartments—the matrix and the intermembrane space (IMS) (2).

Mitochondrial membranes have a dynamic architecture, which is enabled by a continuous supply of lipids and proteins. While some lipids, like cardiolipin (CL), are synthesized within the organelle from its precursor phosphatidic acid, mitochondria predominantly acquire lipids from the endoplasmic reticulum (ER). Moreover, contact sites facilitating lipid exchange between mitochondria and lipid droplets (LDs), have also been suggested (3, 4). Once imported, these lipids need to be redistributed between the two membranes, but the exact mechanism for this is still unknown. Alterations in the mitochondrial contact site complexes have been shown to disturb mitochondrial ultrastructure and disrupt cristae morphology, suggesting a potential contribution to lipid metabolism (5, 6, 7). Further, lipid transfer proteins mediating a constant exchange of precursor lipids between the MIM and MOM, by shuttling them across the IMS have also been reported (8, 9).

The IMS is a small and rather crowded subcompartment, housing ∼50 proteins in yeast and humans (10, 11). While these numbers account for the soluble protein population, there is also a considerable fraction of MIM and MOM proteins that protrude into the IMS. As a buffer between the two membranes, the IMS allows for exchange of lipids, metabolites, and movement of proteins to maintain mitochondrial function. These proteins are typically small and are imported through either the disulfide relay pathway or the stop-transfer pathway. Additionally, some proteins such as CytC, Prx1, and Adk1 to name a few, are imported through atypical pathways some of which are yet to be fully elucidated (12, 13).

Tgl2 is a TAG lipase localized to the mitochondrial IMS (14, 15). It belongs to the Tgl family of lipases in *Saccharomyces cerevisiae* of which Tgl1, Tgl3, Tgl4, and Tgl5 can be found in LDs or cytosol (16). A previous study suggested lipolytic activity of Tgl2 toward short-chain triacylglycerides (TAGs) and diacylglycerides (DAGs) and long-chain TAGs (14). The protein has also been shown to compensate for the absence of DAG Kinase in *Escherichia coli* (17). While deletion of *TGL2* does not result in a detectable phenotype, we previously identified negative genetic interactions of *TGL2* with *MCP2* (15). Elevated levels of Mcp2 appear to maintain lipid trafficking to and from mitochondria in ER mitochondria encounter structure (ERMES) deficient yeast (18), and the protein was more recently suggested to be involved in CoQ mobilization from the MIM (19).

Phosphatidylethanolamine (PE) and CL, which is exclusive to mitochondria, are synthesized in mitochondria from precursor phosphoglycerolipids that not only need to be imported but also have to be transported across the IMS, since certain biosynthesis steps take place in the MIM. PE is synthesized from phosphatidylserine (PS) by the MIM decarboxylase Psd1 (20). CL is assembled from the precursor phosphatidic acid (PA) in the MIM, before maturation of the unique lipid takes place (9). Of the repertoire of IMS proteins, the only known evolutionarily conserved lipid transfer system is the Ups1/Ups2/Mdm35 complex (9, 21, 22, 23). This system is involved in the trafficking of the aforementioned lipids PA and PS (9, 20, 22, 23). Therefore, and since neutral lipids like TAGs are primarily stored in and mobilized from LDs, reports on the presence of a putative TAG lipase in the IMS are of high interest.

In this study, we characterized Tgl2 and studied its quality control. We identified its putative catalytic site and investigated its effect on cellular neutral lipid content.

## Results

### Tgl2 forms a homodimer that is sensitive to reducing agents

**Figure 1:**
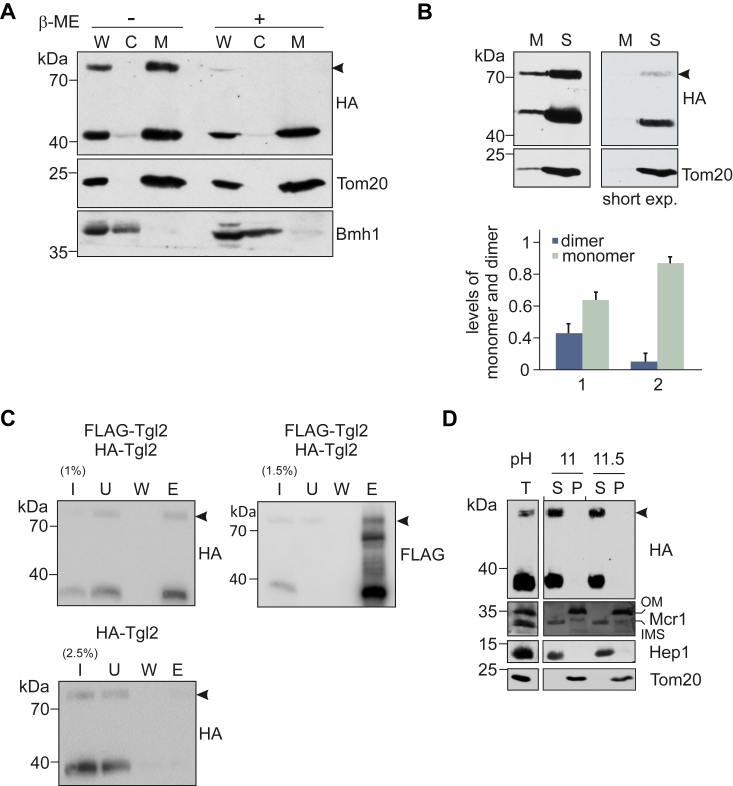
**Tgl2 forms a homodimer that is sensitive to reducing agents.***A*, Tgl2 forms a higher molecular weight adduct under nonreducing conditions. Cells lacking *TGL2* (*tgl2*Δ) were transformed with a plasmid encoding HA-Tgl2. Mechanical lysis was used to isolate whole cell lysate (W), cytosolic (C), and mitochondrial (M) fractions. The samples were mixed with sample buffer in the absence (−) or presence (+) of beta-mercaptoethanol and analyzed by SDS-PAGE and immunodecoration with antibodies against the HA-tag, as well as Tom20 (mitochondrial marker) and Bmh1 (cytosolic marker). The high molecular weight adduct is marked by an *arrowhead*. *B*, the higher MW species is more pronounced upon shorter isolation protocols. Cells expressing HA-Tgl2 were grown to mid-logarithmic phase and mitochondrial fractions were isolated afterward either mechanical lysis (M) or spheroplasting (S). The samples were analyzed by nonreducing SDS-PAGE and immunodecoration. Quantification of the high MW species was done with Tom20 as a loading control and the total Tgl2 amounts were taken as 100%. The graph represents the mean values ± SD of four independent experiments. *C*, the high MW species is a homodimer of Tgl2. Mitochondria were isolated from *tgl2*Δ cells expressing either only HA-Tgl2 or coexpressing HA-Tgl2 and FLAG-Tgl2. The organelles were solubilized and subjected to coimmunoprecipitation with anti-FLAG beads. Samples from input (I), unbound (U, 5%), wash (W, 5%), and eluate fractions (E, 50%) were analyzed by SDS-PAGE and immunodecoration with antibodies against either the HA- or the FLAG-tag. *D*, Tgl2 dimer is soluble. Isolated mitochondria containing HA-Tgl2 (total, T) were subjected to alkaline extraction at different pH values. The supernatant (S) and pellet (P) fractions were analyzed by SDS-PAGE and immunodecoration with the indicated antibodies. Tom20, MOM protein; Hep1, soluble matrix protein; Mcr1, a protein with two isoforms, long form embedded in the mitochondrial OM and shorter soluble form in the IMS. IMS, intermembrane space; MOM, mitochondrial outer membrane.

Tgl2 harbors eight cysteine residues that are essential for its stability and import through the Mia40 pathway (15). Since all our previous studies on Tgl2 were performed under reducing conditions, we asked whether the protein would behave differently under nonreducing conditions, especially since the IMS has a more oxidizing environment than the cytosol or matrix (24). We isolated crude mitochondria from yeast cells overexpressing N terminally hemagglutinin (HA) tagged Tgl2 (HA-Tgl2) and omitted reducing agents during SDS-PAGE. A higher molecular weight species, approximately twice the size of Tgl2, was observed (Fig. 1*A*). Of note, this dimer-like form was not detected when a reducing agent beta-mercaptoethanol was added to the sample (Fig. 1*A*). Further, this species disappeared upon longer incubation periods. It could be detected more evidently in whole cell lysates and crude mitochondria isolated after rather short mechanical rupturing but to a lesser extent in mitochondria obtained from spheroplasting-based longer isolation protocols (Fig. 1*B*). We further quantified this higher molecular weight species using the MOM protein Tom20 as a loading control. We observed that after shorter isolation protocols, this species contributed almost half of the total protein amount whereas its appearance was noticeably reduced upon longer isolation protocols (Fig. 1*B*). Therefore, we assume that the species is not a consequence of oxidation, which would be expected to increase over time.

Since the size of this higher molecular weight species is twice that of Tgl2, we assumed that it could be a homodimer of Tgl2. To test our hypothesis, we isolated mitochondria from cells coexpressing two variants of Tgl2–N terminally tagged with either HA- or FLAG-tag and performed coimmunoprecipitation with beads containing antibodies against the FLAG-tag. We analyzed the fractions by Western blotting and immunodecoration with antibodies against the two different tags. HA-tagged Tgl2 was detected only in the eluate fraction from mitochondria containing both tagged proteins. As a control, we could verify that the anti-FLAG beads do not unspecifically interact with HA-Tgl2. Furthermore, we could also detect the higher molecular weight species of both variants in the eluate fraction (Fig. 1*C*, arrowhead). Taken together, the two tagged Tgl2 species are indeed interacting with each other, and the higher molecular species is most likely a homodimer.

The submitochondrial localization of Tgl2 was previously determined through hypoosmotic lysis assays, and we previously showed by alkaline extraction that monomeric Tgl2 is not embedded in a mitochondrial membrane (15). However, we wondered if varying the pH could alter the response of either the monomeric or the dimeric form of the protein to alkaline extraction. To that end, isolated mitochondria were subjected to alkaline extraction assay under different pH values and the pellet and supernatant fractions were analyzed by SDS-PAGE followed by immunodecoration with antibodies against Tom20, Mcr1, and Hep1 as controls for membrane integrated and soluble proteins (Fig. 1*D*). We observed that the dimeric form is also found in the soluble fraction under both pH conditions, indicating that both forms of the protein are soluble.

### Tgl2 is associated with the mitochondrial inner membrane

**Figure 2:**
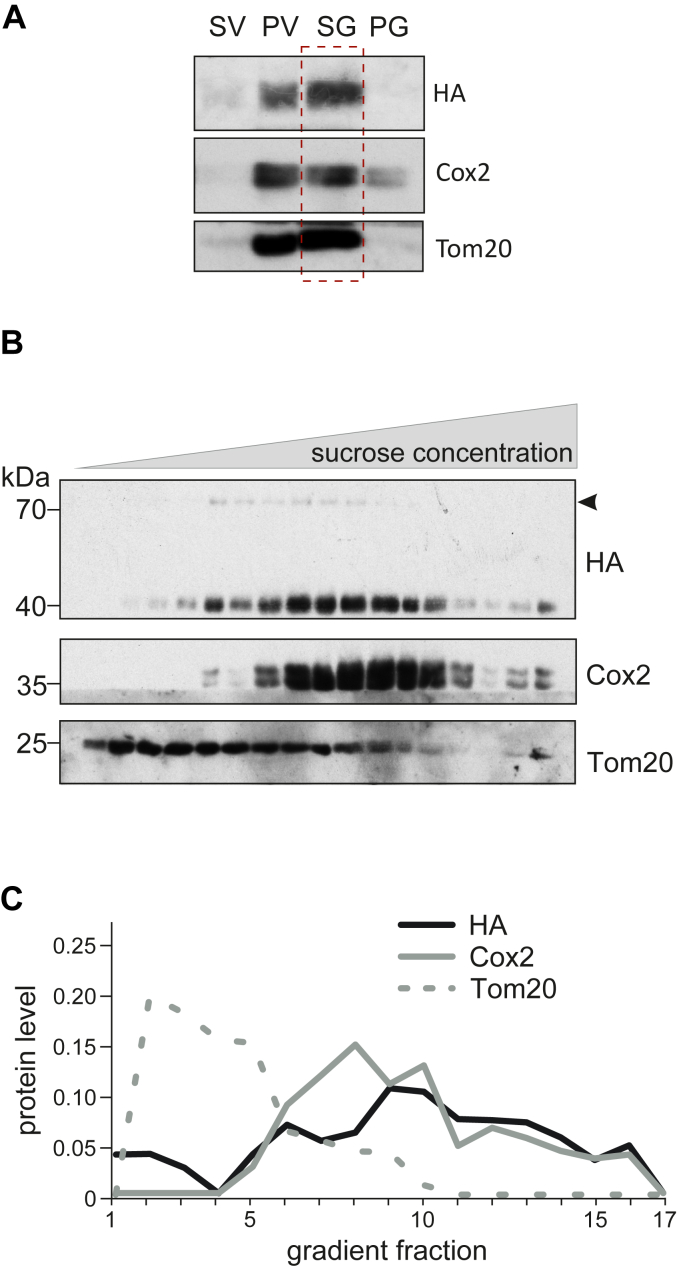
**Tgl2 associates with the mitochondrial inner membrane.***A*, the majority of Tgl2 is found in mitochondrial vesicles. Mitochondrial vesicles were obtained after swelling and sonication of mitochondria isolated from cells containing HA-Tgl2. The sample was sonicated to form vesicles followed by clarifying spin to yield supernatant (SV) and pellet (PV) fractions. The vesicles in PV were sonicated again and subjected to a slower clarifying spin resulting in SG (supernatant) and PG (pellet) fractions. The fractions were analyzed by SDS-PAGE and immunodecoration. Cox2, a MIM protein. *B*, Tgl2 associates with MIM vesicles. The mitochondrial vesicles from SV fraction (described above) were separated by sucrose density centrifugation. Fractions were collected and analyzed by SDS-PAGE and immunodecorated with the indicated antibodies. *C*, quantification (average) of n = 3 independent experiments as shown in (B). Tgl2 and Cox2 containing fractions coincide. Intensities of the bands corresponding to Tgl2, Cox2, and Tom20 were quantified and normalized to the total content of each protein. MIM, mitochondrial inner membrane.

A protein like Tgl2, with a suggested affinity for lipids, would need to associate with at least one of the mitochondrial membranes to gain access to potential substrates. We asked whether we could observe a specific interaction of Tgl2 with vesicles representing one of the mitochondrial membranes. To ascertain this, we performed a sucrose gradient centrifugation with mitochondrial vesicles containing HA-Tgl2 (Fig. 2*A*). The fractions of the gradient were analyzed by Western blotting. Tgl2 was found in vesicles of higher density that also contained the *bona fide* MIM protein Cox2 and was almost completely absent in lighter fractions marked by the presence of the MOM protein Tom20 (Fig. 2, *B* and *C*). These observations suggest that the vast majority of Tgl2 tends to associate with the MIM.

### Tgl2 is a component of a higher molecular weight complex

We previously identified *TGL2* in a synthetic genetic array as a negative interactor of *MCP2*- encoding the kinase Mcp2, an IMS protein involved in mitochondrial lipid and coenzyme Q homeostasis (15, 18, 19). However, although we tried several different experimental approaches, we could not observe any physical interaction between Tgl2 and Mcp2 (data not shown). Furthermore, coimmunoprecipitation experiments with tagged Tgl2 and subsequent proteomic analysis failed to reveal Mcp2 as a putative interactor (Table S1). Of note, no other prominent putative interaction partner(s) could be identified through pull-down assays followed by mass spectrometry (representative experiment provided as Table S1). This analysis revealed as hits, ribosomal proteins, and abundant mitochondrial proteins such as Porin and Om45. The dataset also contained numerous abundant MIM proteins like Mir1, Cox2, and Cyc1 which could be due to the association of the Tgl2 complex with the MIM (Fig. 2*B*).

**Figure 3:**
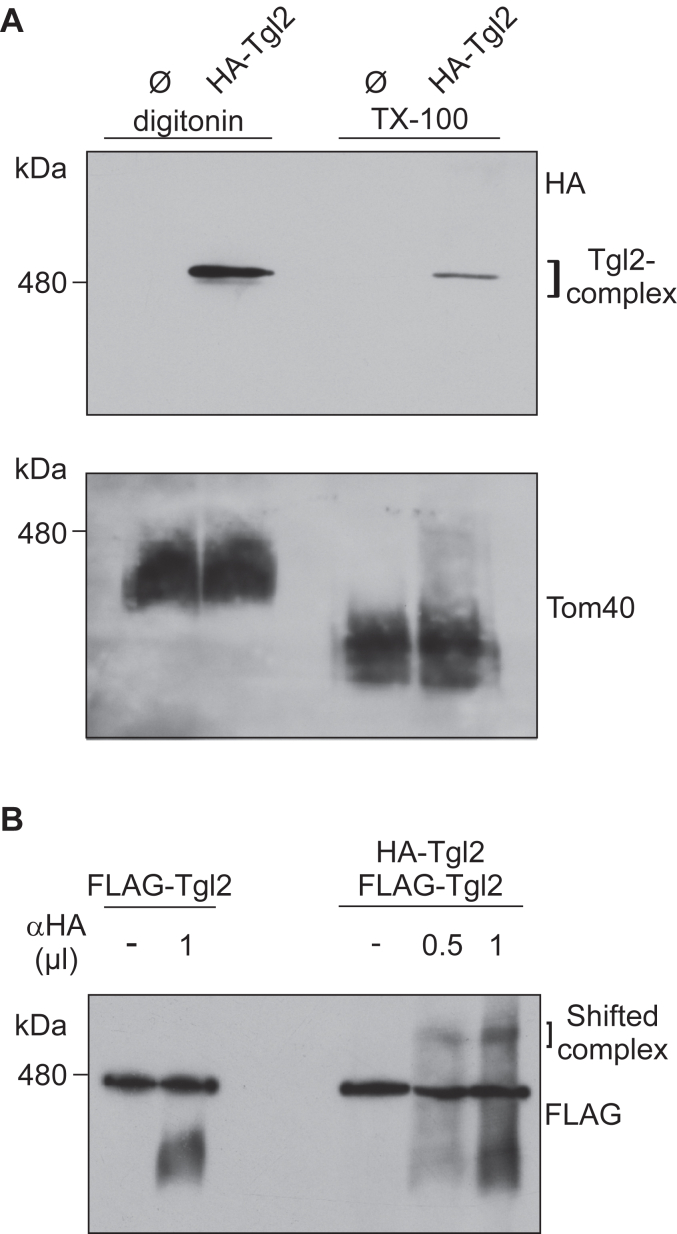
**Tgl2 is a component of a higher molecular weight complex.***A*, Tgl2 forms a higher molecular weight complex. Mitochondria containing HA-Tgl2 were solubilized with either digitonin or TritonX-100 (Tx-100) and subjected to BN-PAGE followed by immunodecoration with antibodies against either HA-tag or Tom40, as a loading control. *B*, Tgl2 complex contains at least two copies of the protein. Mitochondria containing either only HA-Tgl2 or HA-Tgl2 and FLAG-Tgl2 were solubilized with digitonin. The lysate was incubated with 0, 0.5, or 1 μl of anti-HA antibody (α-HA) with a concentration of 100 μg/ml and analyzed by BN-PAGE and immunodecoration with anti-FLAG antibody. BN, blue native.

Even though we could not identify promising putative interactors, we wanted to know if Tgl2 forms a higher molecular weight complex. To address this, we isolated mitochondria from yeast overexpressing HA-Tgl2, solubilized them with either digitonin or Triton X-100, and subjected them to blue native (BN)-PAGE followed by immunodecoration. We observed that Tgl2 migrates as part of a complex of 500 kDa that was detected in higher amounts upon mild solubilization using digitonin (Fig. 3*A*). As expected, no signal was detected in cells transformed with an empty vector verifying that the anti-HA antibody does not display any cross-reactivity. This approach was also used to analyze the Tgl2 complex in yeast strains deleted for proteins that according to their function and/or location in the IMS might be potential interactors of Tgl2. Table S2 provides an overview of the different deletion mutants analyzed and the roles of the respective proteins in mitochondrial physiology. However, we did not observe a change in size or loss of the Tgl2-complex in any of these mutants (Fig. S1*A*). We also monitored the steady state levels of monomeric and dimeric Tgl2 in the various deletion strains and the dimeric protein was always detected (Fig. S1*B*). Of note, the Tgl2 complex appears as a sharp solitary band unlike known mitochondrial membrane embedded complexes like TOM complex that displays a rather broad band in native gels (Fig. 3*A*). The sharp band observed for the Tgl2-complex in BN-PAGE is in agreement with a recent high-throughput complexome study (Schulte *et al.*, 2023) where Tgl2 is present as a rather sharp peek of similar molecular weight. This behavior further suggests that Tgl2 is indeed a soluble protein of defined quaternary structure without associated membrane lipids.

We next asked if the Tgl2-containing complex contains multiple copies of the protein. To address this, we performed an antibody shift assay with solubilized mitochondria containing one or two differently tagged variants of Tgl2. Prior to the BN-PAGE analysis we incubated the mitochondrial lysate with an antibody against the HA-tag and immunodecorated against the FLAG-tag. We observed a size shift only for the sample containing both tagged Tgl2 variants (Fig. 3*B*). As expected, the complex was not shifted when mitochondria containing only FLAG-Tgl2 were used. These observations indicate that the complex contains at least two copies of Tgl2.

### The MIM protease Yme1 mediates the quality control of Tgl2

**Figure 4:**
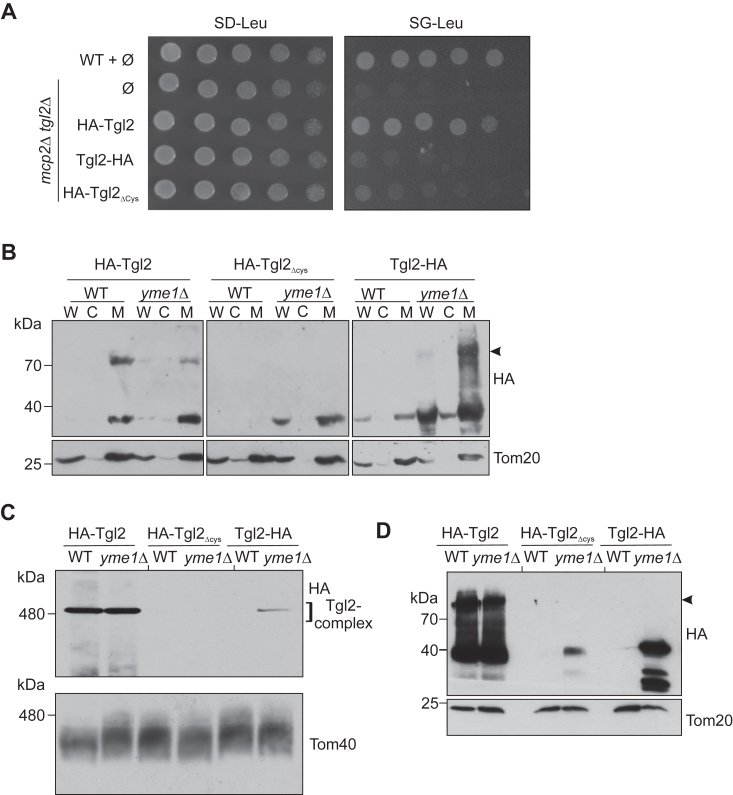
**Yme1 mediates the quality control of Tgl2.***A*, C terminally tagged Tgl2 and Tgl2 variant with all its cysteines mutated to serine are not functional. WT (W303-1A) or *mcp2*Δ *tgl2*Δ cells were transformed with an empty vector (∅) or a plasmid encoding the indicated Tgl2 variant. Cells were analyzed by drop-dilution assay on glucose (SD-Leu) or glycerol (SG-Leu) containing plates at 30 °C and imaged after 5 days. *B*, WT (W303-1A) or *yme1*Δ cells encoding the indicated Tgl2 variants were grown on galactose containing medium and whole cell (W), cytosolic (C), or crude mitochondrial (M) fractions were isolated. The samples were analyzed by SDS-PAGE and immunodecoration with antibodies against HA and Tom20 as a loading control. The high molecular weight species is marked by an *arrowhead*. *C*, mitochondria isolated from either WT (W303-1A) or *yme1*Δ cells expressing the indicated Tgl2 variants were solubilized with digitonin and analyzed by BN-PAGE followed by immunodecoration with antibodies against HA or Tom40, as a loading control. *D*, aliquots (5%) of the solubilized mitochondria described in (*C*) were taken after the clarifying spin and analyzed by SDS-PAGE and immunodecoration with antibodies against HA and Tom20, as a loading control. BN, blue native.

Previous studies regarding quality control of IMS proteins have reported that Yme1—an iAAA protease of the MIM, is responsible for degrading misfolded or unstable proteins in the IMS (25, 26). Many of these proteins are also substrates of the disulfide relay import pathway. We previously observed that C terminally modified Tgl2 and Tgl2_ΔCys_, whose eight cysteine residues were mutated to serine, were hardly detectable in WT yeast cells. These protein variants were also unable to rescue the growth defect exhibited by the *mcp2*Δ *tgl2*Δ double mutant (15). To test whether nonfunctional and potentially misfolded Tgl2 variants are substrates of Yme1, we compared the steady state levels of N terminally tagged Tgl2 (HA-Tgl2), C terminally tagged Tgl2 (Tgl2-HA), and HA-Tgl2_Δcys_ in cells lacking endogenous Tgl2 to those in *yme1*Δ cells. We previously reported that only a functional variant of Tgl2 can rescue the growth phenotype exhibited by *mcp2Δ tgl2*Δ, and consequently used this as a readout to assess the functionality of the Tgl2 variants. We performed a drop-dilution assay on nonfermentable carbon sources (glycerol) and could confirm these observations (Fig. 4*A* and (15)). A stark growth defect was observed for *mcp2Δ tgl2*Δ on glycerol-containing medium which could only be rescued by introducing HA-Tgl2 but not the other two variants, further confirming that Tgl2-HA and HA-Tgl2_Δcys_ are nonfunctional. To compare the steady state levels of these variants, we obtained whole cell lysate, cytosolic, and crude mitochondrial fractions from either *tgl2*Δ or *yme1*Δ cells expressing these protein variants, and analyzed them by Western blotting (Fig. 4*B*). We could detect the native-like HA-Tgl2 in *tgl2*Δ as well as *yme1*Δ cells. However, Tgl2-HA and HA-Tgl2_Δcys_ were only detected in the fractions from *yme1*Δ cells. These findings strongly support the notion that Yme1 is involved in the elimination of nonfunctional variants of Tgl2. Interestingly, HA-Tgl2_Δcys_ is unable to form a homodimer, strengthening the hypothesis that the observed dimer is indeed formed by disulfide bridges.

Next, we wanted to analyze if nonfunctional Tgl2 variants form higher molecular weight complexes, and if these complexes behave similar to the native one. We isolated mitochondria from *tgl2*Δ and *yme1*Δ cells expressing the variants of interest, solubilized them with digitonin, and analyzed them by BN-PAGE and immunodecoration (Fig. 4*C*). We could detect the complex for HA-Tgl2 in mitochondria isolated from both *tgl2*Δ and *yme1*Δ cells. In contrast, a complex containing Tgl2-HA could be detected, although at highly reduced levels, only in mitochondria lacking Yme1 (Fig. 4*C*). Of note, HA-Tgl2_Δcys_ is unable to form a complex even in the absence of Yme1 indicating that the complex is dependent on cysteine residues. As a control, the levels of the TOM complex, detected with an antibody against Tom40, are not altered in the relevant strains (Fig. 4*C*). To correlate the levels of the complex to the overall expression amounts of the TGL2 variants, a fraction of the solubilized mitochondria was loaded also on SDS-PAGE and analyzed using the indicated antibodies. We observed that Tgl2-HA and HA-Tgl2_Δcys_ were detected only in the organelles lacking Yme1 (Fig. 4*D*).

### Analysis of the contribution of single cysteine residues of Tgl2

**Figure 5:**
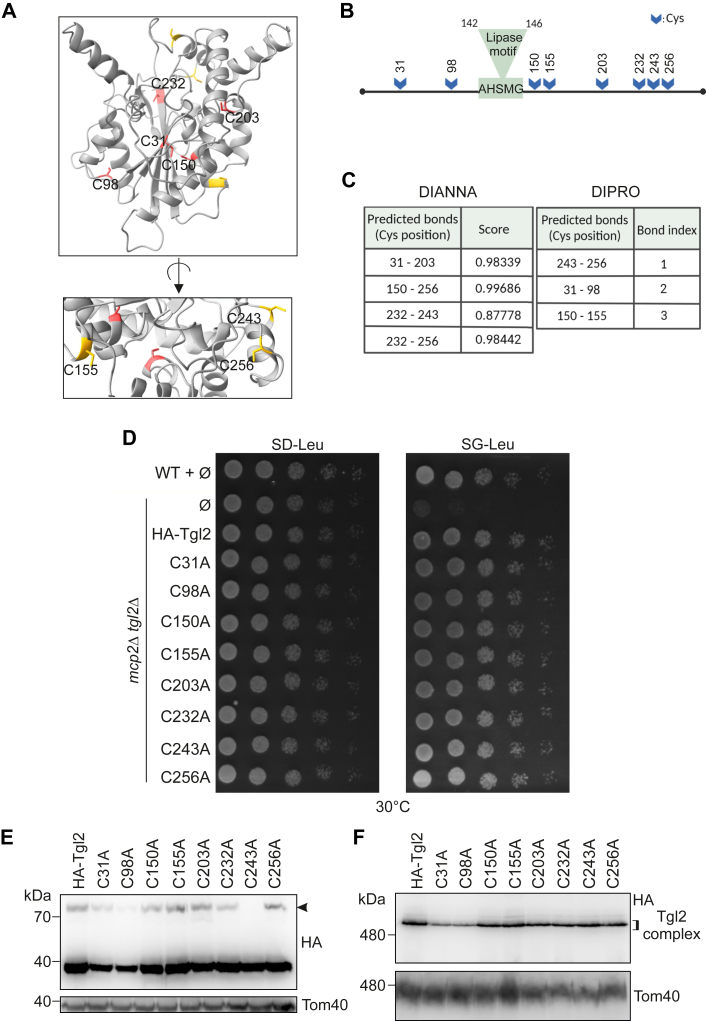
**Analysis of the importance of single cysteine residues of Tgl2.***A*, predicted structure of Tgl2 with the cysteine residues highlighted in *red* (internal residues) or *yellow* (exposed residues). The *upper* panel (internal cysteines lebeled) was rotated so that the exposed cysteines are facing the viewer in the *lower* panel (exposed residues labeled) *B*, schematic representation of the cysteine residues of Tgl2 marked with *blue arrows* and the canonical lipase motif marked in *green* (created with BioRender, https://www.biorender.com/). *C*, prediction of putative disulfide bonds within Tgl2 using either DiANNA (https://bioinformatics.bc.edu/clotelab/DiANNA/) or DIPro (http://scratch.proteomics.ics.uci.edu/). DiANNA scores reflect the probability of disulfide formation. The DIPro bond index is an indication of confidence for disulfide formation (max = 1). *D*, WT (W303-1A) or *mcp2*Δ *tgl2*Δ cells were transformed with an empty vector (∅) or a plasmid encoding the indicated variants. Cells were analyzed by drop-dilution assay on SD-Leu or SG-Leu plates at 30 °C and imaged after several days. *E*, crude mitochondrial fractions were isolated from *tgl2*Δ cells expressing the indicated variants of Tgl2. The samples were analyzed by SDS-PAGE and immunodecoration with the indicated antibodies. *F*, mitochondria from *tgl2*Δ cells expressing the indicated protein variants were solubilized with digitonin and analyzed by BN-PAGE and immunodecoration with antibodies against HA or Tom40. BN, blue native.

The closest structural homologue of Tgl2 is a bacterial lipase–LipA, located in the periplasmic space of *Pseudomonas aeruginosa*. LipA has an intramolecular disulfide bond (between C183 and C235), which is important for the stability of the protein (27, 28). Disruption of this disulfide bridge results in loss of lipolytic function as well as rapid degradation of the protein (27). Since the Tgl2_Δcys_ variant is highly unstable (Fig. 4*B*), we wondered if Tgl2 has any specific intramolecular disulfide bonds that are essential for its stability. Using AlphaFold (https://alphafold.ebi.ac.uk/) for structural prediction of Tgl2, two cysteine residues (C31 and C150) were proposed to potentially form an intramolecular disulfide bridge (Fig. 5, *A* and *B*, (29, 30)). Additionally, we used bioinformatics tools (31, 32, 33) to independently predict the oxidation states of all the cysteine residues and potential disulfide bridges between them (Fig. 5*C*). These predictions and the observation of a dimer under non-reducing condition led us to construct single cysteine mutants of all eight cysteine residues. We replaced each of the cysteines with alanine by site-directed mutagenesis and tagged the variants by HA. Next, we transformed the Cys variants into *tgl2*Δ and *mcp2Δ tgl2*Δ cells. Surprisingly, we observed that all eight single mutants could rescue the growth defect of *mcp2Δ tgl2*Δ (Fig. 5*D*). Thus, it seems that despite the predicted participation of several of the cysteines in disulfide bridges, these cysteine residues are individually not essential for the functionality of the protein.

To study the steady-state levels of the eight mutants, we isolated crude mitochondrial fractions from *tgl2*Δ cells and analyzed them by Western blotting. We observed that the expression levels of most of the cysteine variants were comparable to that of the native protein. A lower expression level was consistently observed for the mutants C31A and C98A (Fig. 5*E*). Both are predicted to face the interior of the protein structure and might form stabilizing disulfide bonds (Fig. 5*A*). Yet, the single Cys mutants are much more stable than the HA-Tgl2_Δcys_ where all eight Cys residues are mutated at once. Most interestingly, we observed that only for the point mutation C243A the dimer was missing (Fig. 5*E*). Indeed, this cysteine residue is predicted to be exposed to the surface of the protein and could be involved in intermolecular disulfide bridges (Fig. 5*A*). Yet, our observation that although the protein is incapable of dimer formation still rescues the growth defect of the *mcp2Δ tgl2*Δ (Fig. 5*D*) suggests that the dimer is not absolutely required for function under the tested conditions.

Since the Tgl2 complex is cysteine dependent (Fig. 4*C*), we wanted to analyze if any of the individual cysteine residues is essential for its formation and/or stability. With that aim, we analyzed by BN-PAGE isolated mitochondria harboring the various cysteine variants of Tgl2 (Fig. 5*F*). We could not observe any noteworthy difference in the complex formation or stability between the native protein and the cysteine mutants. It seems that the lower levels of the complex in organelles harboring either C31A or C98A variants can be explained by the overall lower levels of these variants (compare Fig. 5, *E* and *F*). Thus, we conclude that these single-point mutations by themselves are not sufficient to destabilize either the homodimer or oligomer formed by Tgl2, and therefore have no obvious impact on the functionality of the protein.

### The predicted catalytic triad of Tgl2 is required for its functionality

**Figure 6:**
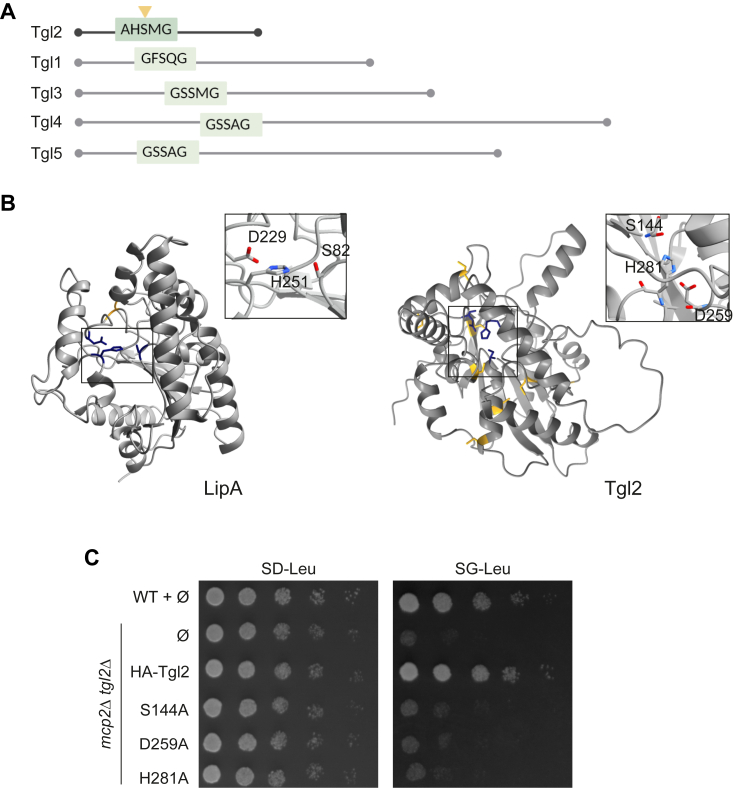
**The predicted catalytic triad of Tgl2 is required for its functionality.***A*, schematic presentation of members of the yeast Tgl family. The characteristic lipase motif is indicated in *green* with a catalytically active serine with *yellow arrow*. Adapted from (16). *B*, the predicted catalytic triad of Tgl2 (Ser-Asp-His). The atomic structure of LipA (crystal structure 1EX9, PDB, *left*) was used to predict the catalytic site of Tgl2 (*right*). The intramolecular disulfide bond of LipA is highlighted in *yellow* and the catalytic triad consisting of S82, D229, and H251 is highlighted in *blue* (28, 37) The cysteine residues of Tgl2 are indicated in *yellow* and the predicted catalytic triad (S144-D259-H281) in *blue*. *C*, WT (W303-1A) or *mcp2*Δ *tgl2*Δ cells transformed with the indicated variants were analyzed by drop-dilution assay on either SD-Leu or SG-Leu plates at 30 °C and imaged after several days. PDB, Protein Data Bank.

Tgl2 was first categorized as a lipase due to its canonical lipase motif - (G/A)XSXG, of which the serine is the catalytically active residue (Fig. 6*A*, adapted from (16)). In *in vitro* lipase assays using affinity tag-enriched protein, Ham *et al.* reported that mutating serine 144 to alanine (HA-Tgl2_S144A_) leads to loss of lipolytic activity of Tgl2 (14). Moreover, prokaryotic and eukaryotic lipases typically contain a catalytic triad consisting of Ser-Asp-His, wherein the serine is often part of the conserved (G/A)XSXG motif. This triad is also analogous to the catalytic center of serine proteases as well as acyltransferases such as Tgl3 and Lro1 ((34, 35, 36) and Fig. S3). We used structural data on LipA as well as other bacterial lipases that share homology with Tgl2, to gain more insight into the catalytic triad of Tgl2 (37). *In silico* analysis of the predicted structure of Tgl2 suggest a catalytic triad comprising of Ser144, Asp259, and His281 (Fig. 6*B*) (29, 30, 38).

To test the importance of the amino acids that build the predicted catalytic triad, we mutated to alanine the catalytically active S144 (14), as well as H281 or D259. Next, we tested the ability of the resulting variants to complement the growth phenotype exhibited by *mcp2Δ tgl2*Δ cells. As expected, we could observe comparable growth behaviors of all the tested strains on fermentable carbon sources. However, in agreement with the previous reports, HA-Tgl2_S144A_ was unable to complement the growth phenotype exhibited by *mcp2Δ tgl2*Δ on glycerol-containing medium (Fig. 6*C*). Similarly, both HA-Tgl2_H281A_ and HA-Tgl2_D259A_ were also unable to rescue the growth phenotype under these conditions. These findings indicate that residues S144, H281, and D259 are most likely involved in the catalytic triad of the protein.

### Disruption of the catalytic triad leads to protein instability

**Figure 7:**
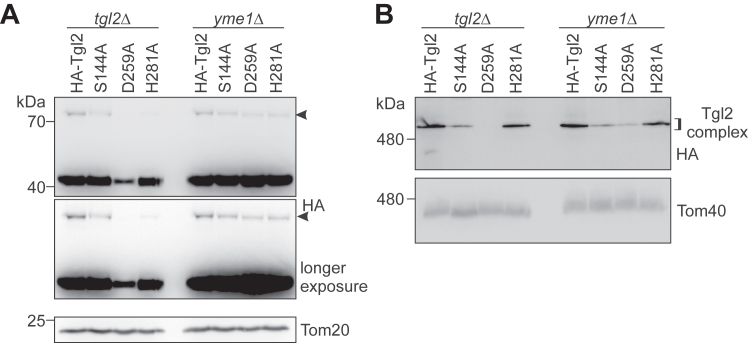
**Disruption of the catalytic triad leads to protein instability.***A*, crude mitochondrial fractions were isolated from *tgl2*Δ and *yme1*Δ cells expressing the indicated Tgl2 variants. The samples were analyzed by SDS-PAGE and immunodecoration with antibodies against HA and Tom20, as a loading control. The high molecular weight species is marked by an *arrowhead*. *B*, mitochondria isolated from the cells described in (*A*) were solubilized with digitonin and analyzed by BN-PAGE and immunodecoration with antibodies against HA and Tom40. BN, blue native.

Considering the nonfunctionality of the variants with mutated catalytically triad, we wondered how these mutations affect the stability and oligomerization of Tgl2. Monitoring the steady-state levels of the different variants we noticed that only the one mutated in D259 was expressed to lower levels on *tgl2*Δ background. Interestingly, the deletion of *YME1* rescued the expression levels (Fig. 7*A*), suggesting that HA-Tgl2_D259A_ is unstable and therefore removed by Yme1.

Next, we were interested to investigate the oligomeric state of these mutants. Figure 7*B* shows that the complex containing the mutated Ser144 could be detected in lower amounts and replacing D259 resulted in an even further compromised complex amounts. Of note, deleting the protease *YME1* did not improve the oligomerization state of these two variants (Fig. 7*B*), supporting the notion that the highly reduced levels of the complex are not due to overall reduced amounts of the protein. In contrast to the other two residues of the catalytic triad, it seems that His281 is crucial neither for the overall stability of the protein nor for its oligomerization capacity (Fig. 7).

These observations are in congruence with previous studies where the catalytically active aspartate residue was shown to be crucial for protein stability in lipases from *Geotrichum candidum* as well as human pancreatic lipase and choline esterase. These previous studies have also reported that while mutating the serine and histidine residues in the catalytic triad leads to loss of function, they have no impact on protein stability (39).

### Genetic interactions of *MCP2*, *TGL2*, and *YME1*

In this study, we assess several different mutants of Tgl2 to gain a better understanding of the protein’s structure–function relationship. We use complementation of *mcp2 Δtgl2*Δ under nonfermentable conditions as a tool to analyze functionality. Of the mutants created in this study, the nonfunctional variants (HA-Tgl2_Δcys_, Tgl2-HA, HA-Tgl2_S144A,_ HA-Tgl2_H281A,_ and HA-Tgl2_D259A_) either could not be detected or were detected in very low amounts in *mcp2Δ tgl2*Δ (Fig. S2). Naturally, this raises the question whether these variants are indeed nonfunctional due to loss of enzymatic capacity or are unable to complement the growth phenotype simply due to their low expression levels.

**Figure 8:**
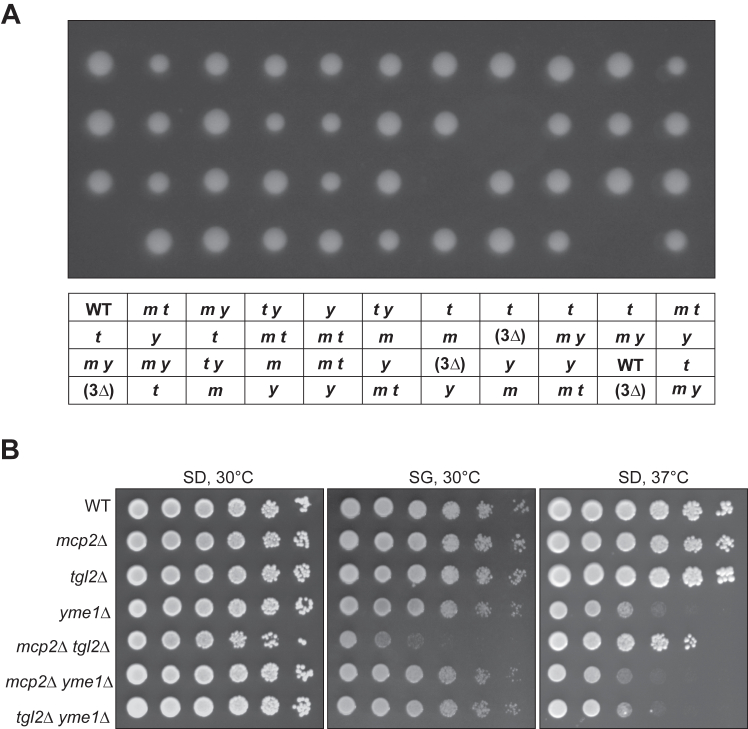
**Genetic interactions of *MCP2*, *TGL2*, and *YME1*.***A*, excerpt of 11 out of more than 100 dissected tetrads of the heterozygous triple deletion mutant (*mcp2Δ/MCP2 - tgl2Δ/TGL2 - yme1Δ/YME1*). The table below the haploid clones shows the genotype analysis, *m*: *mcp2Δ*, *t*: *tgl2Δ*, *y*: *yme1Δ*, WT: wild type (W303-1A), (3*Δ*): triple deletion–not viable. *B*, growth analysis of single and double deletion strains of the three genes. Growth at either 30 °C or 37 °C was analyzed by drop dilution assay on synthetic medium containing either glucose (SD) or glycerol (SG).

Since we could show that Yme1 is responsible for the turnover of Tgl2 (Fig. 4*B*), we attempted to create the triple mutant strain *mcp2Δ tgl2Δ yme1*Δ to ensure that the mutated Tgl2 variants are not degraded and the growth defect caused by the absence of Mcp2 and Tgl2, can be analyzed in the absence of Yme1. Interestingly, the deletion of all three genes always yielded nonviable spores. Figure 8*A* shows an excerpt from n > 100 tetrads dissected.

Of note, we observed that lethality of the triple deletion mutant does not stem from a simple combination of growth defects caused by the three individual genes. All three single deletion mutant cells as well as all combinations of double mutations show no growth defect on glucose containing synthetic medium at 30 °C. Furthermore, the growth defect of *yme1*Δ cells at elevated temperatures (37 °C) is not worsened by the additional loss of either Mcp2 or Tgl2. Finally, on nonfermentable carbon sources where *mcp2*Δ *tgl2*Δ cells show the pronounced growth defect, *yme1*Δ cells grow like WT (Fig. 8*B*).

Although we so far cannot explain the cause of lethality of the combined deletion of *MCP2*, *TGL2*, and *YME1*, we assume that all three proteins share a functional relationship. As expected, when we express plasmid borne functional Tgl2 in heterozygous diploid triple mutant cells prior to tetrad dissection we can retrieve triple deletion cells expressing plasmid borne Tgl2. On the other hand, empty plasmids or plasmids encoding the nonfunctional Tgl2 variant Ser144A failed to yield triple deletion cells expressing the mutant proteins (data not shown).

### Mitochondria lacking Tgl2 have elevated levels of neutral lipids

**Figure 9:**
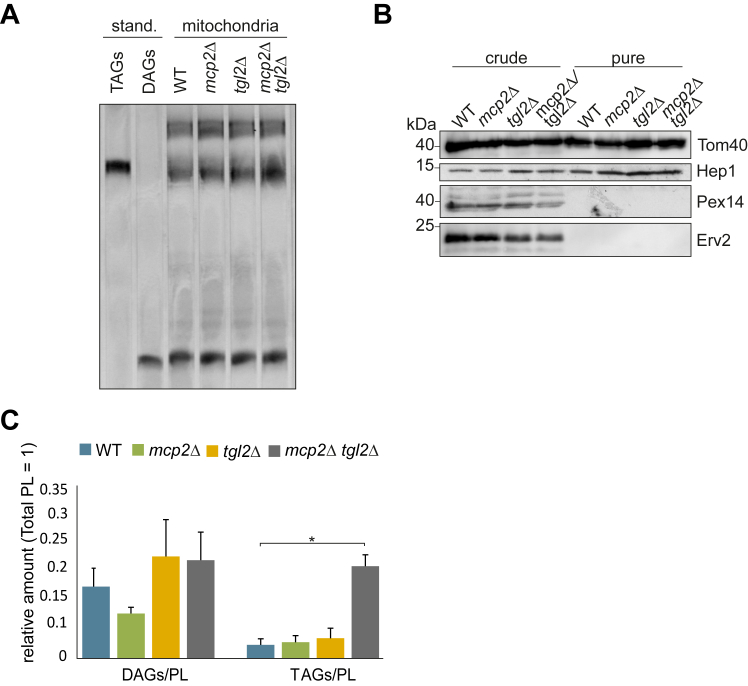
**Mitochondrial fractions from *mcp2*Δ *tgl2*Δ cells show increased TAG levels.***A*, neutral lipids were extracted from mitochondria isolated from the indicated cells and analyzed by thin-layer chromatography. The lipids were stained with a primuline solution and visualized under UV-light. The migration behavior of standard neutral lipids is indicated on the *left*. *B*, crude mitochondria were isolated from the indicated strains, treated with trypsin and further purified by sucrose gradient. Crude and purified mitochondria (100 μg) were analyzed by SDS-PAGE and immunodecoration. Tom40 and Hep1, mitochondrial markers; Pex14, peroxisomal marker; and Erv2, ER marker. *C*, pure mitochondria from (*B*) were analyzed by mass spectrometric analysis. The amounts of TAGs and DAGs *versus* total phosphoglycerolipids (PL) is shown as a mean with SD bars (n = 3, ∗*p* < 0.05). DAG, diacylglyceride; TAG, triacylglyceride.

Previous lipidomics analysis from whole cell extracts indicated elevated TAG and DAG levels in *mcp2Δ tgl2*Δ yeast cells (15). Since Tgl2 and Mcp2 are mitochondrial proteins, we wondered if this perturbation is primarily caused by mitochondrial alternations. To that goal, we extracted phosphoglycerolipids and neutral lipids from mitochondria isolated from control and mutated cells (WT, *mcp2*Δ*, tgl2*Δ, and *mcp2Δ tgl2*Δ) and subjected them to TLC analysis. As in whole cell lipidomic analysis reported before (15), the phosphoglycerolipid pattern was similar for all tested mitochondrial extracts (Fig. S4). On the other hand, we observed a slight increase in TAG levels in isolated mitochondria from *mcp2Δ tgl2*Δ cells (Fig. 9*A*). To improve the purity of our mitochondrial samples and further analyze the lipid composition of the isolated organelles, we trypsinized the mitochondria from WT, *mcp2*Δ*, tgl2*Δ, and *mcp2Δ tgl2*Δ, to eliminate protein-mediated mitochondrial contact sites, and purified the organelles by a density gradient. The purity of the mitochondria was confirmed by SDS-PAGE and immunodecoration with antibodies against peroxisomal (Pex14), ER (Erv2), and mitochondrial (Tom40 and Hep1) marker proteins. Figure 9*B* confirms that the pure mitochondrial fractions are essentially free from ER and/or peroxisome contaminations. Lipidomics analyses of the purified mitochondria revealed elevated TAG and DAG levels in organelles from *mcp2Δ tgl2*Δ cells (Fig. 9*C*). Collectively, these results suggest that the combined deletion of *TGL2* and *MCP2* results in an increase in mitochondrial or mitochondria-associated TAGs.

## Discussion

The importance of cellular lipid homeostasis is highlighted by its implication for human health (40, 41). Even in simpler model organisms like *Saccharomyces cerevisiae*, deficiencies in lipid metabolism result in impaired cellular function and, in some cases, severe growth defects (40, 41). There have been significant advancements in the field of lipid metabolism in the last few years wherein the crosstalk between mitochondria and other organelles has acquired a crucial role. Reports of protein tethers between the MOM and membranes of proximal organelles laid the foundation of the concept of “contact sites”, which has become a topic of extensive research. The ERMES complex was the first of such mitochondrial tethers to be recognized, and has been now thoroughly elucidated (42, 43, 44). ERMES subunits have been shown to bind to and traffic phospholipids between the ER and mitochondria. The loss of these subunits results in severe growth phenotypes and anomalous mitochondrial morphology (45, 46, 47, 48).

In a previous study, we characterized a high copy suppressor of ERMES mutants–Mcp2 (now Cqd2) which was more recently suggested to be involved in CoQ metabolism in yeast (19). To better characterize *MCP2/CQD2*, we performed a high throughput screen of *mcp2Δ* and identified *TGL2* as a hit, where the two genes displayed negative genetic interaction, more prominently on nonfermentable carbon sources (15). This growth defect is lost after prolonged growth on fermentable carbon sources—a phenomenon often observed for lipid metabolism defects and ERMES mutants (45, 49, 50). We could also show that both Mcp2 and Tgl2 are located in the IMS. Of the two, Tgl2 is a soluble protein and relies on the disulfide relay pathway for its import into the IMS (15).

In our current study, we further characterized Tgl2 to gain a better understanding of its molecular structure and function. We observed that Tgl2 can form homodimers under nonreducing conditions and suggest that the dimer is a product of intermolecular disulfide bridges between cysteine 243 of each monomer of the protein. Although unlikely, we cannot rule out, that the disulfide bridge is formed with an IMS protein of approximately the same size as Tgl2. Notably, our extensive pull-down and mass spectrometric analyses did not identify such an interactor. We could also confirm an oligomeric form of the protein containing at least two copies of Tgl2. This complex is also thiol dependent and is susceptible to treatment with stronger detergents. The formation of a structural important disulfide bridge would agree with a more oxidizing environment in the IMS, similar to the periplasm of bacteria. Interestingly, Tgl2 is not conserved among higher eukaryotes and has its closest structural homologues in prokaryotes. Such bacterial lipases *e.g.* LipA from *P. aeruginosa*, were shown to harbor intramolecular as well as intermolecular disulfide bridges. Furthermore, extracellular lipases of higher eukaryotes like human pancreatic lipase, and lipoprotein lipase also contain disulfide bonds which are often essential for protein function, allowing dimerization that facilitates substrate binding or protein stability (27, 51, 52). Since mutating all cysteine residues of Tgl2 led to loss of function of the protein, we aimed to pinpoint which of the eight cysteine residues is important for stability and/or function of the protein. To that aim, we mutated all cysteine residues individually therefore covering all potential intramolecular or intermolecular cysteine bridges. However, replacing each of the single cysteines to alanine had no influence on the quaternary structure or function of Tgl2. These findings suggest that more than one internal cysteine-bridge is present in native Tgl2, and loss of a single disulfide bond has only a minor effect on the stability of the protein. Indeed, the mutated variants C31A and C90A were expressed to a lower amount than the other six. Future structural studied might shed light on this issue.

The nonfunctional variants of Tgl2 like Tgl2-HA and Tgl2_Δcys_, are nearly undetectable in WT or *tgl2Δ* cells despite the aim to overexpress them, indicating a strict quality control mechanism for the protein. A vast majority of the IMS proteins rely on Yme1, an i-AAA protease in the MIM, for their folding and/or degradation (26). Yme1 has an IMS exposed catalytic domain which is suggested to have also chaperone-like activity (25). By comparing the steady state levels of native and nonfunctional variants of Tgl2, we could confirm that its quality control is maintained by Yme1. The undetectable or weakly detectable protein mutants could be observed upon deletion of *YME1* in amounts similar to those of native Tgl2. We also found a synthetic lethal genetic interaction of *MCP2*, *TGL2*, and *YME1* which suggests a functional relationship beyond the quality control of Tgl2 by Yme1. Further studies are required to understand this observation.

Ham *et al.* previously characterized the lipase motif of Tgl2. By mutating the conserved serine residue (S144), they could show a loss of *in vitro* lipolytic activity (14). Our observations that despite native-like expression levels, Tgl2_S144A_ is unable to rescue the growth defect of *mcp2*Δ *tgl2*Δ, agree with their report. Of interest is our finding that this variant displays less oligomerization as compared to the native protein suggesting a correlation between activity and the formation of the high molecular weight complex. We also characterized the predicted Ser-His-Asp catalytic triad of Tgl2 and showed that disrupting the catalytically active triad by replacing the aspartate by alanine leads to an unstable, poorly expressing variant of the protein. In contrast to D259, replacing the His-residue (H281) did not affect the stability of the protein although it also culminated in nonfunctional variant. Studies pertaining to other lipases and acyltransferases show that while a conservative mutation (aspartate to glutamate) results in only partial loss of function, mutating the aspartate to alanine or valine leads to loss of enzyme activity and stability. Since the carboxylic group is essential for stabilizing interactions with other residues in its vicinity, its loss can lead to protein misfolding and/or instability (36, 39). The catalytic triad is a feature of several enzymes including proteases, acyltransferases, and lipases (Fig. S3 and (51)). Some members of the Tgl family exhibit lipase as well as acyltransferase activity (53, 54), therefore Tgl2 could also be involved in transferring fatty acids between substrates rather than hydrolyzing TAGs like classical lipases Of note, the closest structural homologue of Tgl2 is a bacterial TAG lipase–LipA, which is located in the periplasm. Given the bacterial ancestry of mitochondria and the similarity of Tgl2 with mostly bacterial lipases, one could speculate that during the course of evolution, the protein was maintained in simpler eukaryotes like yeast but was lost in higher organisms.

The lipolytic activity of Tgl2 was shown previously by *in vitro* lipase assays wherein Tgl2 enriched lysate was used to hydrolyze different TAGs—of which tributyrin was the optimal substrate (14). Of note, tributyrin is not a typical physiological substrate in mitochondria. We performed lipidomics analysis of purified mitochondrial fractions and could detect elevated levels of neutral lipids in fractions isolated from *mcp2*Δ/*tgl2*Δ yeast cells. This observation aligns with our previous lipidomics analysis from whole cell lysate fractions of the same cells (15). Notably, the neutral lipid content measured in the lipidomics analyses reflected primarily long-chain fatty acids, which are the most abundant forms of TAGs and DAGs in yeast cells. In conclusion, our experiments could show that the combined absence of Tgl2 and Mcp2 results in increased neutral lipid amounts in yeast cells which is mainly caused by an increase in TAGs in mitochondrial fractions. Further experiments are required to clarify whether TAGs indeed occur in between the leaflets of either the MIM or the MOM. We cannot exclude that stronger interaction between LDs and mitochondria, in the absence of Mcp2 and Tgl2, leads to an increased LD cosedimentation during isolation of mitochondria. The physiological function of Tgl2 in the IMS is currently unclear, and further studies are required to confirm whether it behaves as a classical TAG lipase or it evolved to perform a different role.

To achieve a comprehensive understanding of Tgl2’s function, we tried to identify its physical interaction partners through coimmunoprecipitation experiments followed by mass spectrometry experiments. Despite using different affinity tags and experimental conditions, we were unable to find any proteins of interest in these assays. In a recent mitochondrial complexome study, Tgl2 was shown to have a similar migration behavior as Ups1 and Ups3, LTPs in the IMS (55). To investigate potential Tgl2-Ups1 interactions, we performed BN-PAGE with *ups1*Δ yeast cells expressing HA-Tgl2 but the complex remains unchanged in size and stability (Fig. S1). We applied the same approach to *ups2*Δ and *mdm35*Δ, proteins involved in the lipid transfer complex of the IMS (22, 23) but the complex was still unchanged (Fig. S1). These findings support the notion that the Tgl2-containing complex is probably a homooligomer.

Taken together, our study sheds new light on the structure–function relationship of the mitochondrial protein Tgl2.

## Experimental procedures

### Yeast strains and growth conditions

Yeast strains were grown in standard rich medium (YP) or synthetic medium (S) with either glucose, galactose, or glycerol as the carbon source.

Transformation of yeast cells was done using the lithium acetate method. For strains transformed with plasmids, synthetic media with appropriate selection marker(s) were used.

For drop dilution assays, yeast cells were cultured to an *A*_600_ of 1.0 and serially diluted in five-fold increments. Aliquots (5 μl) of the serially diluted cultures were spotted onto the corresponding solid medium, and the cells were incubated at either 30 or 37 °C.

All deletion strains were made using tetrad dissection and confirmed by PCR using primers specific for the genes of interest.

All *S. cerevisiae* strains used in this study are listed in Table 1.

**Table 1:** *Saccharomyces cerevisiae* strains used in this study

Name	Genotype	Reference
W303-1A	MAT a; *ade2-1; can1-100; his3-11; leu2-3112; trp1Δ2; ura3-52*	(65)
W303-1B	MAT α; *ade2-1; can1-100; his3-11; leu2-3112; trp1Δ2; ura3-52*	(65)
YKD432	W303-1A; *mcp2*Δ::HIS3MX6	(66)
YKD898	W303-1A; *tgl2*Δ:: KanMX4	(15)
YKD1009	W303-1A; *mcp2*Δ:: HIS3MX6; *tgl2*Δ::KanMX4	(15)
YKD552	W303-1A; *yme1*Δ:: KanMX6	Gift from T. Langer
YKD492	W303-1A; *gep4*Δ:: KanMX4	(15)
YKD472	W303-1A; *psd1*Δ:: KanMX4	(15)
YTT158	W303-1A; *ups2*Δ::Nat	(22)
YTT159	W303-1A; *ups1Δ*::Nat	(22)
YKD806	W303-1A; *mdm35Δ*::HIS3MX6	Lab stock
YKD180	W303-1A; *mdm31Δ*::KANMX4	(46)
YKD182	W303-1A; *mdm32Δ*::HIS3MX6	(46)
YKD175	W303-1A; *fzo1Δ*::HIS3MX6	Lab stock
BY4741	MATa his3Δ1 leu 2Δ0 met15Δ0 ura3Δ0	Lab stock
YMS306	BY4741;*om45Δ*::KANMX4	Euroscarf
YKD986	W303-1A; *tgl1Δ*::KANMX4	Lab stock
YKD991	W303-1A; *tgl3Δ*::KANMX4	Lab stock
YDP038	W303-1A; *fis1Δ*::KANMX4	Lab stock
YFO002	W303-1A; rho^0^	Lab stock
YDA283	BY4741; *crd1Δ*::HIS3MX6	Euroscarf
YDA107	BY4741; *taz1Δ*::KANMX4	Euroscarf

### Recombinant DNA techniques

The previously constructed plasmid pGEM-Tgl2 (15) was used as a template for inserting a FLAG tag at the N terminus of the ORF of Tgl2. The construct pGEM-HA-Tgl2 was created using primers from (15) and this construct served as a template for site-directed mutagenesis reactions. Primer pairs containing the mutation of interest were used to generate a mutated sequence of Tgl2. The PCR products were digested using DpnI and transformed into *E. coli* cells for further screening. After confirmation upon DNA sequencing, the mutated constructs were cloned into the yeast expression plasmid pYX142.

All primers and plasmids used in this study are listed in Tables 2 and 3, respectively.

**Table 2:** Primers used in this study

Name	Sequence	Remarks
FLAG-Tgl2-For	GGGGAATTCATGGACTACAAAGACGATGACGACAAG AAAAATGATAATAAAGCTAATGATATAATAATA	Insert FLAG tag at the N terminus of Tgl2
Tgl2_S144A_-For	CTAATCGCACACGCAATGGGGGGAC	Substitution of Ser (TCA) to Alanine (GCA) at position 144
Tgl2_S144A_-Rev	GTCCCCCCATTGCGTGTGCGATTAG	
Tgl2_D259A_-For	GGTTGCCCCAACGCTGGCCTTGTAACCATA	Substitution of Asp (GAT) to Alanine (GCT) at position 259
Tgl2_D259A_-Rev	TATGGTTACAAGGCCAGCGTTGGGGCAACC	
Tgl2_H281A_-For	GGACTTTGAAGGACATGGATGCCCTGGACGTCATCAATTGGAA	Substitution of His (CAT) to Ala (GCC) at position 281
Tgl2_H281A_-Rev	TTCCAATTGATGACGTCCAGGGCATCCATGTCCTTCAAAGTCC	
Tgl2_C31A_-For	AAAAATCCTATTGTATTTGCCCATGGTTTATCAGGATTT	Substitution of Cys (TGC) to Ala (GCC) at position 31
Tgl2_C31A_-Rev	AAATCCTGATAAACCATGGGCAAATACAATAGGATTTTT	
Tgl2_C98A_-For	AATTTCTTCAATCTAAGGGAGCCACTGTTATCACCACTAAGGT	Substitution of Cys (TGT) to Ala (GCC) at position 98
Tgl2_C98A_-Rev	ACCTTAGTGGTGATACAGTGGCTCCCTTAGATTGAAGAAATT	
Tgl2_C150A_-For	ACTCAATGGGGGGACTAGACGCACGATATCTAATTTGCAATAT	Substitution of Cys (TGC) to Ala (GCA) at position 150
Tgl2_C150A_-Rev	ATATTGCAAATTAGATATCGTGCGTCTAGTCCCCCCATTGAGT	
Tgl2_C155A_-For	TAGACTGCCGATATCTAATTGCAAATATAAAAAATAGGAATTA	Substitution of Cys (TGC) to Ala (GCC) at position 155
Tgl2_C155A_-Rev	TAATTCCTATTTTTTATATTTGCAATTAGATATCGGCAGTCTA	
Tgl2_C203A_-For	GCCAAAAGATATTGCCAATAGCCTTCTACCAACTCACGACTGC	Substitution of Cys (TGT) to Ala (GCC) at position 203
Tgl2_C203A_-Rev	GCAGTCGTGAGTTGGTAGAAGGCTATTGGCAATATCTTTTGGC	
Tgl2_C232A_-For	TCTTATTTTTCGTATGGAGCCTCCTTTGTGCCTAAGTG	Substitution of Cys (TGC) to Ala (GCC) at position 232
Tgl2_C232A_-Rev	CACTTAGGCACAAAGGAGGCTCCATACGAAAAATAAGA	
Tgl2_C243A_-For	TAAGTGGTACAATGTCTTTGCCACTCCCTGGAAAATTGTTT	Substitution of Cys (TGT) to Ala (GCC) at position 243
Tgl2_C243A_-Rev	AAACAATTTTCCAGGGAGTGGCAAAGACATTGTACCACTTA	
Tgl2_C256A_-For	AGGTCTAAAGGTGCCCCCAACGATGGCCT	Substitution of Cys (TGC) to Ala (GGC) at position 256
Tgl2_C256A_-Rev	AGGCCATCGTTGGGGGCACCTTTAGACCT

**Table 3:** List of plasmids used in this study

Name	Promoter	Markers	Coding sequence
pYX142-HA-Tgl2	TPI	LEU2, Amp^R^	Tgl2 with N-terminal HA-Tag
pYX142-Tgl2-HA	TPI	LEU2, Amp^R^	Tgl2 with C-terminal HA-Tag
pYX142-HA-Tgl2_Δcys_	TPI	LEU2, Amp^R^	Tgl2 with N-terminal HA-Tag, all cysteines mutated to serine
pGEM4-HA-Tgl2	T7	Amp^R^	Tgl2 with N-terminal HA-Tag
pYX122-FLAG-Tgl2	TPI	HIS3, Amp^R^	Tgl2 with N-terminal FLAG-Tag
pYX142-HA-Tgl2_S144A_	TPI	LEU2, Amp^R^	Tgl2 with N-terminal HA-Tag, S144A mutation
pYX142-HA-Tgl2_D259A_	TPI	LEU2, Amp^R^	Tgl2 with N-terminal HA-Tag, D259A mutation
pYX142-HA-Tgl2_H281A_	TPI	LEU2, Amp^R^	Tgl2 with N-terminal HA-Tag, H281A mutation
pYX142-HA-Tgl2_C31A_	TPI	LEU2, Amp^R^	Tgl2 with N-terminal HA-Tag, C31A mutation
pYX142-HA-Tgl2_C98A_	TPI	LEU2, Amp^R^	Tgl2 with N-terminal HA-Tag, C98A mutation
pYX142-HA-Tgl2_C150A_	TPI	LEU2, Amp^R^	Tgl2 with N-terminal HA-Tag, C150A mutation
pYX142-HA-Tgl2_C155A_	TPI	LEU2, Amp^R^	Tgl2 with N-terminal HA-Tag, C155A mutation
pYX142-HA-Tgl2_C203A_	TPI	LEU2, Amp^R^	Tgl2 with N-terminal HA-Tag, C203A mutation
pYX142-HA-Tgl2_C232A_	TPI	LEU2, Amp^R^	Tgl2 with N-terminal HA-Tag, C232A mutation
pYX142-HA-Tgl2_C243A_	TPI	LEU2, Amp^R^	Tgl2 with N-terminal HA-Tag, C243A mutation
pYX142-HA-Tgl2_C256A_	TPI	LEU2, Amp^R^	Tgl2 with N-terminal HA-Tag, C256A mutation

### Isolation of mitochondria

Mitochondria were isolated from yeast cells using a previously published protocol based on differential centrifugation (56). Yeast cultures (2–5 L) were grown at 30 °C and harvested at an *A*_600_ of 0.8 to 1.6 by centrifugation (3000*g*, 5 min, room temperature (RT)). The cell pellets were washed, weighed, and resuspended in resuspension buffer (100 mM Tris and 10 mM DTT). The cell suspension was incubated at 30 °C for 10 min after which the cells were harvested as above. The cell pellets were washed with a spheroplasting buffer (1.2 M sorbitol, 20 mM sodium phosphate, pH 7.2). Next, the cell pellets were resuspended in zymolyase-containing spheroplasting buffer and incubated at 30 °C for at least 1 h with constant shaking. The spheroplasts were harvested (1100*g*, 5 min, 2 °C), resuspended in homogenization buffer (0.6 M sorbitol, 1 mM EDTA, 1 mM PMSF, 0.2% (w/v) fatty-acid free bovine serum albumin, 10 mM Tris, pH 7.4), and dounced 10 to 12 times. The lysed spheroplasts were first subjected to a clarifying step (2000*g*, 5 min, 2 °C) and then to a high-speed centrifugation step (17,500*g*, 15 min, 2 °C). The pellets were washed with the isotonic SEM buffer (250 mM sucrose, 1 mM EDTA, 10 mM Mops/KOH, pH 7.2) containing 2 mM PMSF and centrifuged as in the previous step. The pellet obtained after the high-speed spin is the mitochondrial fraction. The pellet was resuspended in SEM buffer, snap-frozen in liquid N_2_, and stored at −80 °C until further use.

To obtain pure mitochondrial fractions, the mitochondrial pellet obtained after differential centrifugation was subjected to trypsinization (5 μg of trypsin per mg of mitochondrial protein) on ice for 20 min. The reaction was stopped by adding soybean trypsin inhibitor (10 μg soybean trypsin inhibitor per μg of trypsin) and incubated on ice for 30 min. The trypsinized mitochondria were mixed with 2 to 3 ml of SEM buffer containing 1 mM PMSF and loaded on a sucrose step gradient (20, 30, 40, 50, and 60% (w/v) sucrose in 10 mM Mops/KOH pH 7.4, 100 mM KCl, 1 mM EDTA, and 1 mM PMSF). The gradients were centrifuged (210,000*g*, 16 h, 4 °C) using a swing-out rotor (SW40Ti). The two fractions between the 40% and 60% phase—the mitochondrial fractions were collected and diluted with 35 ml SEM buffer containing 2 mM PMSF. A pure mitochondrial pellet was harvested by centrifuging the suspension (17,500*g*, 15 min, 4 °C). The pellet was resuspended in SEM buffer, snap-frozen, and stored at −80 °C until further use.

### Alkaline extraction

Isolated mitochondria (50 μg) were resuspended in 50 μl of 20 mM Mops/KPH pH 7.5. Next, 50 μl of 0.2 M sodium carbonate solution of varying pH (11 or 11.5) was added to the samples to a final concentration of 0.1 M, which were then incubated on ice for 30 min. The samples were centrifuged (94,000*g*, 30 min, 4 °C), and the pellet was resuspended in sample buffer. The supernatant was subjected to trichloroacetic acid precipitation, and the resulting pellet was also resuspended in sample buffer. The samples were boiled at 95 °C for 10 min and analyzed by SDS-PAGE and immunodecoration.

All antibodies used in this study are listed in Table 4.

**Table 4:** List of antibodies used in this study

Antibody	Dilution	Source
Polyclonal rat anti-HA	1:1000	Roche
Polyclonal rabbit anti-Erv2	1:1000	Lab of Roland Lill
Polyclonal rabbit anti-Hep1	1:2000	Lab stocks
Polyclonal rabbit anti-Pex14	1:10,000	Lab stocks
Polyclonal rabbit anti-Bmh1	1:10,000	Lab stocks
Polyclonal rabbit anti-Tom20	1:4000	Lab stocks
Polyclonal rabbit anti-Tom40	1:4000	Lab stocks
Polyclonal rabbit anti-Tom70	1:2000	Lab stocks
Polyclonal rabbit anti-FLAG	1:2000	Thermo Fischer Scientific
Horseradish peroxidase-coupled goat anti-rabbit	1:10,000	1721019 (Bio-rad)
Horseradish peroxidase-coupled goat anti-rat	1:5000	ab6845 (Abcam)

### Isolation of crude mitochondria by mechanical rupture

Yeast cultures (100–200 ml) were grown at 30 °C and harvested at an *A*_600_ of 0.8 to 1.6 by centrifugation (3000*g*, 5 min, RT). The pellet was resuspended in SEM buffer, and the cells were disrupted by repeated cycles of vortexing with glass beads at 4 °C. The lysed cells were centrifuged (2000*g*, 3 min, 4 °C) to remove unbroken cells and the crude mitochondria were pelleted from the supernatant by centrifugation (14,000*g*, 12 min, 4 °C).

### Co-immunoprecipitation

Mitochondria (1 mg) were reisolated and solubilized in 500 μl of Tris-buffered saline (TBS) supplemented with protease inhibitor cocktail and 1% (w/v) digitonin. The lysed mitochondria were incubated on an orbital shaker for 30 min at 4 °C and the solubilized material was centrifuged (18,000*g*, 30 min, 4 °C). Magnetic anti-FLAG beads were incubated with the supernatant on an orbital shaker for 1 h at 4 °C. The beads were washed three times with 500 μl of TBS supplemented with protease inhibitor cocktail, and the bound proteins were eluted using FLAG peptide (150 ng/μl in TBS).

### BN-PAGE

Mitochondria (50 μg) were solubilized in 50 μl of SEM buffer supplemented with either Triton X-100 (0.5% (v/v)) or digitonin (1% (w/v)). The solution was incubated for 30 min at 4 °C on an orbital shaker and the solubilized fraction was subjected to a clarifying spin (20,000*g*, 15 min, 4 °C). The supernatant was mixed with the loading dye (5% (w/v) Coomassie blue G, 500 mM 6-amino-N-caproic acid, 100 mM Bis-Tris, pH 7.0) and analyzed on a 4 to 14% acrylamide blue native gel. The gels were blotted onto a polyvinylidene fluoride membrane and further analyzed by immunodecoration.

### Antibody shift assay

Mitochondria (50 μg) were resuspended in 50 μl of SEM buffer supplemented with 1% digitonin and incubated on an orbital shaker for 30 min at 4 °C. The solubilized mitochondria were subjected to a clarifying spin (20,000*g*, 30 min, 4 °C), and the supernatant was incubated with 1 μl of SEM buffer (as control) or with either 0.5 μl or 1 μl of anti-HA antibody. The samples were incubated on ice for 30 min and then centrifuged briefly (13,000*g*, 10 min, 4 °C). The supernatant was mixed with the loading dye as above and analyzed by BN-PAGE and immunodecoration.

### Extraction and analysis of mitochondrial neutral lipids

Lipid extraction of isolated mitochondria was done according to a published protocol (57).

Briefly, 500 μg of mitochondria were resuspended in a mixture of chloroform:methanol 1:2 (v/v), vortexed, and incubated on ice for 30 min. Next, 1.25 volumes of chloroform followed by 1.25 volumes of water were added—the samples were vortexed vigorously after each addition. The organic and inorganic phases were separated by centrifuging the samples (1000*g*, 10 min, RT). The lower, organic phase was collected with a Pasteur pipette and the samples were evaporated by SpeedVac. The lipids were resuspended in 30 μl of chloroform:methanol 1:1 (v/v) and immediately used for TLC.

Neutral lipids or phosphoglycerolipids were separated by TLC using glass plates of precoated 0.25 mm silica gel with fluorescent indicator UV_254_. The silica plates were prepared by washing them with a mixture of chloroform:methanol 1:1 (v/v) and then allowing them to dry for 15 to 20 min. The plates were then sprayed with a solution of 2.3% boric acid (w/v) in ethanol and dried in an oven for 15 min at 100 °C.

The lipids were spotted gradually (3–5 μl at a time) until the entire volume was spotted onto the TLC plate. The spots were air dried and then separated by a solvent mixture of either chloroform/ethanol/water/trimethylamine (30:35:7:35, v/v/v/v) for phosphoglycerolipids or hexane:diethylamine:acetic acid (70:30:1, v/v/v) for neutral lipids. After the samples have completely migrated through the whole distance, the plate was air dried, and the run was repeated to ensure successful separation. The plate was allowed to dry completely and then sprayed with a 0.05% (w/v) primuline solution in 80% (v/v) acetone. The plate was visualized under a UV-lamp.

### Sucrose gradient

A sucrose gradient (0.9/1/1.1/1.2/1.3 M) to separate MIM and MOM vesicles was performed according to a previously published protocol (18). Briefly, mitochondria (3 mg) were reisolated and resuspended in 10 ml of swelling buffer (20 mM Hepes/KOH pH 7.4, 2 mM EDTA, and 2 mM PMSF) and incubated for 1 h at 4 °C on an orbital shaker. The sucrose concentration was adjusted to 0.45 M using a high sucrose buffer (2.3 M sucrose, 20 mM Hepes/KOH pH 7.4, 2 mM EDTA, 2 mM PMSF). The suspension was sonicated eight times with 10 s pulses, alternating with a break of 1 min on ice. The sonicated sample was clarified (35,000*g*, 30 min, 4 °C), and the pellet was resuspended in 15 ml of swelling buffer. The solution was centrifuged (200,000*g*, 2 h, 4 °C) to harvest the mitochondrial vesicles. The pellet was resuspended in 400 μl of low sucrose buffer (0.45 M sucrose, 20 mM Hepes/KOH pH 7.4, 2 mM EDTA, and 2 mM PMSF), pipetted up and down several times, and sonicated in a sonifying bath at 4 °C for 5 min.

Next, the sample was spin clarified (35,000*g*, 15 min, 4 °C), and the pellet was resuspended in 1.5 ml of swelling buffer. The supernatant (350 μl) was transferred to a new tube and the sucrose concentration was adjusted to 0.85 M using the sucrose gradient buffer (2.5 M sucrose, 5 mM Mops/KOH, 1 mM EDTA, and 50 mM KCl). The supernatant was loaded onto a sucrose step gradient assembled using the sucrose gradient buffer and the gradient buffer (5 mM Mops/KOH, 1 mM EDTA, and 50 mM KCl) where each layer has a volume of 625 μl. The gradient was centrifuged in a SW60 rotor (230,000*g*, 16 h, 4 °C) such that the lighter MOM vesicles float up while the denser MIM vesicles settle at the bottom. After the centrifugation stepwise 250 μl fractions were collected starting from the top of the gradient, resuspended in sample buffer, and analyzed by SDS-PAGE and immunoblotting.

### Mass spectrometric lipid analysis

Quantitative analysis of lipids was performed by standard nanoelectrospray ionization mass spectrometry (58, 59). Lipids were extracted from 50 μg of yeast mitochondria in the presence of internal standards for the major phospholipid classes and neutral lipids (PC 17:0–14:1, PE 17:0–14:1, PI 17:0–14:1, PS 17:0–14:1, PG 17:0–14:1, 15:0–18:1-d7-PA; CL mix I; d5-TG ISTD Mix I; d5-DG ISTD Mix I; d5-DG ISTD Mix II, all from Avanti Polar Lipids). Extraction was performed according to Bligh and Dyer with some optimization for whole yeast cells (57). Lipids were dissolved in 10 mM ammonium acetate in methanol and analyzed in a QTRAP 6500 triple quadrupole mass spectrometer (SCIEX) equipped with a nanoinfusion spray device (TriVersa NanoMate, Advion). Mass spectra were processed by LipidView Software Version 1.2 (SCIEX) for identification and quantification of lipids. Lipid amounts (pmol) were corrected for response differences between internal standards and endogenous lipids.

### NanoLC-MS/MS analysis and data processing

Coomassie-stained gel pieces were digested in gel with trypsin (60) and desalted peptide mixtures (61) were separated on an Easy-nLC 1200 coupled to a Q Exactive HF mass spectrometer (both Thermo Fisher Scientific) as described elsewhere (62) with slight modifications: peptide mixtures were separated using a 57 min segmented gradient of 10-33 to 50 to 90% of HPLC solvent B (80% acetonitrile in 0.1% formic acid) in HPLC solvent A (0.1% formic acid) at a flow rate of 200 nl/min. In each scan cycle, the seven most intense precursor ions were sequentially fragmented using higher energy collisional dissociation fragmentation. In all measurements, sequenced precursor masses were excluded from further selection for 30 s. The target values for tandem mass spectrometry (MS/MS) fragmentation were 105 charges, and for the MS scan 3 × 106 charges.

Acquired MS spectra were processed with MaxQuant software package version 1.6.7.0 (https://www.maxquant.org/, 63) with integrated Andromeda search engine (64) Database search was performed against a target-decoy *S. cerevisiae* database obtained from Uniprot, containing 6078 protein entries, and 286 commonly observed contaminants.

In database search, full trypsin digestion specificity was required and up to two missed cleavages were allowed. Carbamidomethylation of cysteine was set as fixed modification; protein N-terminal acetylation, and oxidation of methionine were set as variable modifications. Initial precursor mass tolerance was set to 4.5 ppm and 20 ppm at the MS/MS level. A false discovery rate of 1% was applied at the peptide and protein level.

## Data availability

All data is included in the manuscript and the supporting information.

## Supporting information

This article contains supporting information.

## Conflict of interest

The authors declare that they have no conflicts of interest with the contents of this article.

## Supporting information

Suplementary Table S1 Additional Information
