# Supplementary material for: Characterization of the putative yeast mitochondrial triacylglycerol lipase Tgl2
Source: J Biol Chem. 2025 Jan 23;301(3):108217. doi: 10.1016/j.jbc.2025.108217 (PMC11889585; doi:10.1016/j.jbc.2025.108217)
Supplement: Suplementary Table S1 Additional Information [file mmc1.docx]

Supplementary Table S1:

To identify interaction partners of Tgl2, we performed a coimmunoprecipitation assays from isolated mitochondria expressing either FLAG-Tgl2 (labelled R02) or Mim2-Flag (labelled R01) as control for an independent mitochondrial protein. Mitochondria were solubilized using digitonin to retain the Tgl2 complex, and the eluate fractions were analyzed by MS.
