# Supplementary material for: Characterization of the putative yeast mitochondrial triacylglycerol lipase Tgl2
Source: J Biol Chem. 2025 Jan 23;301(3):108217. doi: 10.1016/j.jbc.2025.108217 (PMC11889585; doi:10.1016/j.jbc.2025.108217)
Supplement: Supplementary Table S2 [file mmc3.docx]

Supplementary Table S2:

ORFs of deletion mutants analysed for changes in complex and dimer formation of Tgl2

| **Gene** | **Localization** | **Function** |
| --- | --- | --- |
| *MCP2* | MIM | Lipid homeostasis; CoQ mobilization from the MIM |
| *UPS1* | MIM/IMS | Phosphatidic acid transporter involved in cardiolipin metabolism |
| *UPS2* | IMS | Forms a complex with Mdm35 and transports phosphatidylserine from MOM to MIM; involved in phospholipid metabolism |
| *MDM35* | IMS | Forms a complex with Ups2 and transports phosphatidylserine from MOM to MIM |
| *MDM31* | MIM | Potentially involved in phospholipid metabolism |
| *MDM32* | MIM | Potentially involved in phospholipid metabolism |
| *FIS1* | MOM | Involved in mitochondrial fission |
| *FZO1* | MOM | Involved in mitochondrial fusion |
| *UGO1* | MOM | Involved in mitochondrial fusion by facilitating fusion of MIM and MOM |
| *OM45* | MOM | Unknown function |
| *MIR1* | MIM | Transmembrane phosphate transporter |
| *TGL1* | LDs | Steryl ester hydrolase |
| *TGL3* | LDs | TAG lipase and lysophophatidylethnaolamine acyltransferase |
| *GEP4* | Matrix | Phosphatidylglycerophosphatase, involved in Cardiolipin metabolism |
| *CRD1* | MIM | Cardiolipin synthase, involved in Cardiolipin metabolism |
| *TAZ1* | MOM | Lysolecithin acyltransferase, involved in phospholipid metabolism and cardiolipin remodeling |
| *PSD1* | MIM/ER | Phosphatidylserine decarboxylase, involved in phospholipid metabolism and LD formation |
| *RHO^0^* | - | Yeast strain depleted of mtDNA |
