# Supplementary material for: Characterization of the putative yeast mitochondrial triacylglycerol lipase Tgl2
Source: J Biol Chem. 2025 Jan 23;301(3):108217. doi: 10.1016/j.jbc.2025.108217 (PMC11889585; doi:10.1016/j.jbc.2025.108217)
Supplement: Supplementary Fig. S1 [file mmc4.pdf]

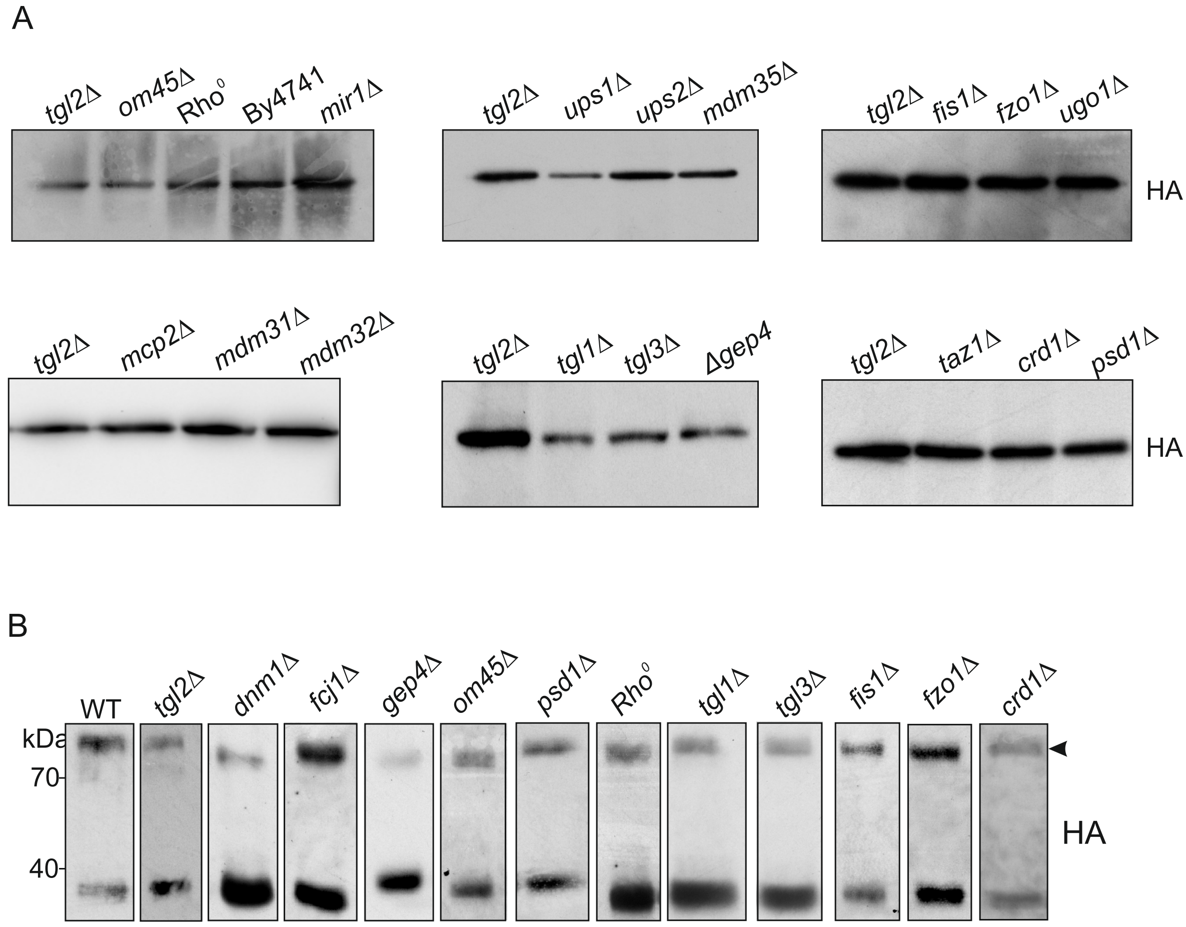


**Fig. S1 Tgl2 complex in different deletion mutants**

(A) The Tgl2 complex is unaffected in the absence of the indicated genes. Mitochondria were isolated from the corresponding deletion strains, solubilized with digitonin, and analysed by BN-PAGE (4-14%) and immunodecoration against HA-tag. There were noticeable effects on the complex formation. (B) Steady state levels of Tgl2 in different deletion strains. Mitochondria were isolated from the indicated strains expressing HA-Tgl2 and analysed by SDS-PAGE and immunodecoration with an antibody against HA-tag. The Western blots are representative of some strains used in this experiment. The intensity of the dimer was quantified with respect to total amount of Tgl2 (n = 3).
