# Supplementary material for: Characterization of the putative yeast mitochondrial triacylglycerol lipase Tgl2
Source: J Biol Chem. 2025 Jan 23;301(3):108217. doi: 10.1016/j.jbc.2025.108217 (PMC11889585; doi:10.1016/j.jbc.2025.108217)
Supplement: Supplementary Fig. S2 [file mmc5.pdf]

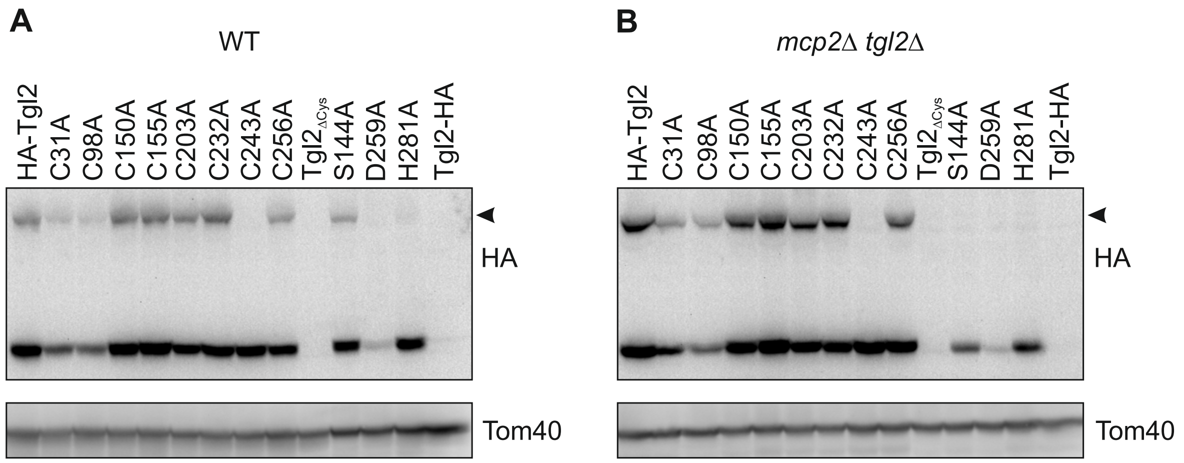

Fig. S2 Steady state levels of Tgl2 mutants

The expression Tgl2 mutants varies in WT (A) and *mcp2*Δ *tgl2*Δ (B) cells. Cells expressing the the indicated variants of Tgl2 were grown to mid-logarithmic phase and crude mitochondrial fractions were isolated. The organelles were analysed by SDS-PAGE and immunodecoration with antibodies against the HA-tag and Tom40, as a loading control. In *mcp2*Δ *tgl2*Δ cells only the functional variants can be detected at comparable levels to the native protein. The non-functional variants are either expressed much less or are not detectable at all.
