# Supplementary material for: Characterization of the putative yeast mitochondrial triacylglycerol lipase Tgl2
Source: J Biol Chem. 2025 Jan 23;301(3):108217. doi: 10.1016/j.jbc.2025.108217 (PMC11889585; doi:10.1016/j.jbc.2025.108217)
Supplement: Supplementary Fig. S3 [file mmc6.pdf]

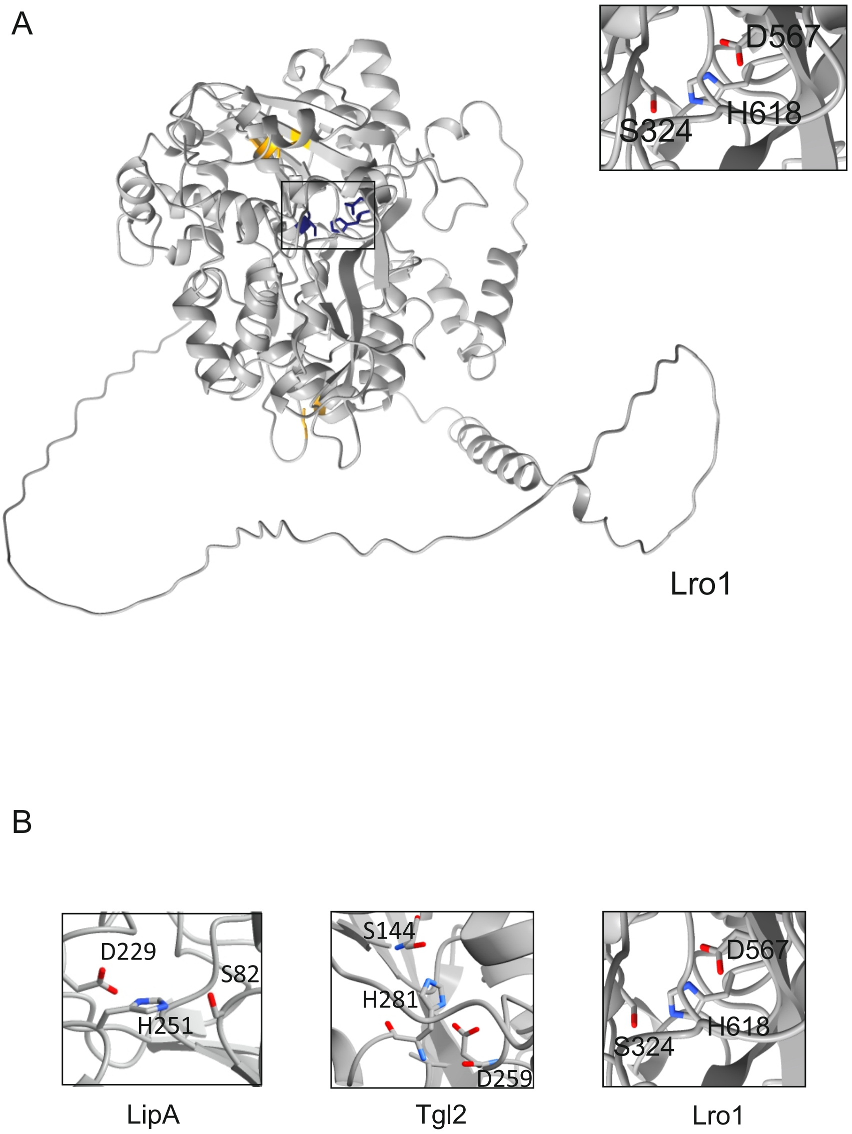

Fig. S3 Catalytic triad of Lro1 – a yeast acyltransferase
(A) Acyltransferases contain a catalytic triad consisting of Ser-Asp-His. Predicted structure of Lro1 with the intramolecular disulfide bonds in yellow and the catalytic triad highlighted in blue. (B) The Ser-Asp-His catalytic triad of LipA (left, a bacterial lipase), Tgl2 (center, yeast putative TAG lipase), and Lro1 (right, yeast acyltransferase).
