# Supplementary material for: Characterization of the putative yeast mitochondrial triacylglycerol lipase Tgl2
Source: J Biol Chem. 2025 Jan 23;301(3):108217. doi: 10.1016/j.jbc.2025.108217 (PMC11889585; doi:10.1016/j.jbc.2025.108217)
Supplement: Supplementary Fig. S4 [file mmc7.pdf]

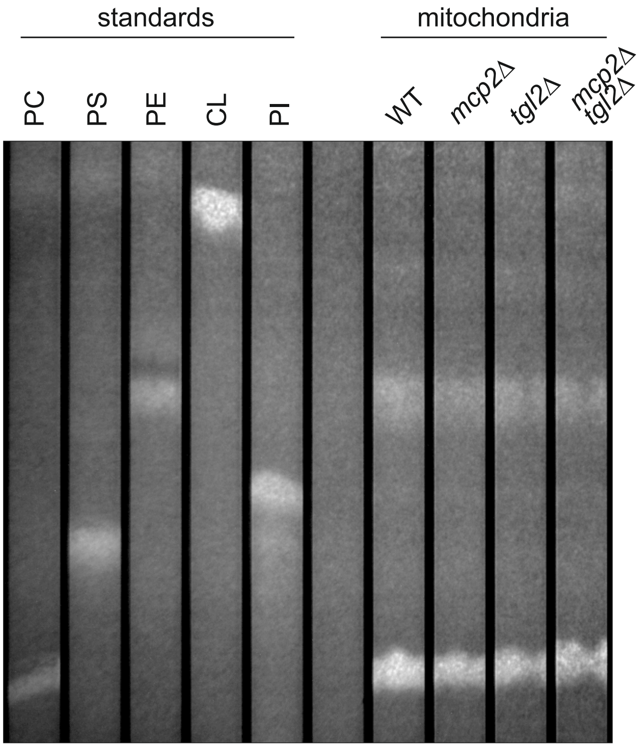

Fig. S4 Phosphoglycerolipids are unaltered in mitochondria lacking Mcp2 and/or Tgl2. Phosphoglycerolipids were extracted from mitochondria of the indicated cells and analysed by thin-layer chromatography. As standards phosphatidylcholine (PC), phosphatidylserine (PS), phosphatidyl-ethanolamine (PE), cardiolipin (CL) and phosphatidylinositol (PI) were loaded. The lipids were stained with a primuline solution and visualised under UV-light.
